# Anti-Apoptotic Gene Delivery with *cyclo*-(d-Trp-Tyr) Peptide Nanotube via Eye Drop Following Corneal Epithelial Debridement

**DOI:** 10.3390/pharmaceutics7030122

**Published:** 2015-07-17

**Authors:** Yu-Hsing Lee, Shwu-Fen Chang, Jiahorng Liaw

**Affiliations:** 1 School of Pharmacy, Taipei Medical University, 250 Wu-Hsing Street, Taipei 11031, Taiwan; E-Mail: m301100020@tmu.edu.tw; 2 Graduate Institute of Medical Sciences, Taipei Medical University, 250 Wu-Hsing Street, Taipei 11031, Taiwan; E-Mail: smbsfc21@tmu.edu.tw

**Keywords:** peptide nanotube, caspase 3, cornea debridement

## Abstract

Corneal keratocyte apoptosis triggered by cornel debridement is one mechanism of corneal disorders. In this study, the feasibility of *cyclo*-(d-Trp-Tyr) peptide nanotubes (PNTs) as carriers of caspase 3 silence shRNA delivery was assessed. A model of epithelial injury by epithelial debridement was applied to investigate the feasibility of PNTs as gene delivery carriers on corneal injury. First, the PNTs were found within 2 μm in length and 300 nm in width by an atomic force microscope and confocal laser microscope system. Plasmid DNAs were observed to be associated with PNTs by atomic force microscope and confocal laser scanning microscope. The plasmids were associated with tyrosine of PNTs with a binding constant of 2.7 × 10^8^ M^−1^. The stability of plasmid DNA with PNTs against the DNase was found at 60 min. Using thioflavin T pre-stained PNTs on the corneal eye drop delivery, the distribution of PNTs was in the epithelial and stroma regions. After corneal debridement, the rhodamine-labeled plasmid DNA and thioflavin T pre-stained PNTs were also delivered and could be observed in the stroma of cornea. PNTs complexed with anti-apoptotic plasmid caspase 3 silencing shRNA eye drop delivery decreased 41% of caspase 3 activity after the first dose by caspase 3 activity and Western blot analysis.

## 1. Introduction

Cornea epithelial debridement often results from cornea surgery, such as laser-assisted *in situ* keratomileusis (LASIK) and photorefractive keratectomy (PRK) in the treatment of myopia, hyperopia or astigmatism [1,2,3]. Cornea epithelial damage is also caused by hypoxia or exposure to alkali, ethanol or UV and even with continued epithelial injury by eye rubbing or contact lens wearing [4,5,6,7]. Loss of keratocytes in stroma was mainly due to cell apoptosis triggered by cornea epithelial damages [1,2,3,8,9,10,11]. In epithelial debridement-induced cornea injury with delayed wound healing, keratocyte apoptosis resulted in the anterior stroma, and subsequent myofibroblast formation determined final visual regression and stromal haze formation, which often occurred after mechanical epithelial scrape and PRK for myopia [1,11,12,13,14]. Keratocyte apoptosis is also involved in the pathophysiology of keratoconus, an ectatic corneal dystrophy that is characterized by progressive thinning of the corneal stroma [7] and the pathogenic mechanisms of aniridia [15]. Thus, anti-apoptosis following corneal damage may reduce the succeeding detrimental effect.

Previously, we used corneal epithelial debridement-induced cornea injury [14,16]. DNA fragmentation was detected at as early as 1 h after epithelial debridement, occurred at the anterior stroma and persisted to 48 h after epithelial debridement. In addition, we found that the protein level of caspase 3 reached a maximum level at 8 and 24 h after debridement [16]. Using topical administration of pCMV-bcl-x_L_-eGFP DNA with sphere-type polymeric micelles (PM) after corneal epithelial debridement, bcl-x_L_-eGFP fusion protein was detected in wounded ocular tissues, and both DNA fragmentation and caspase-3 activity were significantly decreased [16]. Recently, high aspect ratio (AR) particles, such as nanotubes, have drawn attention due to not only their bulk capability [17,18], but also their being taken up in larger amounts with faster internalization rates, as well as prolonged blood circulation time than spherical counterparts [18,19]. Because of the biodegradable and biocompatible properties of amino acids (AAs), increasing interest has focused on linear or cyclic peptides that can self-assemble to form peptide nanotubes (PNTs). Various AA compositions of PNTs provide further surface modification to enhance the interaction with biomembranes, increasing stacking with DNA, forming artificial transmembrane ion channels, *etc.* [20,21,22,23,24], and have a promising usage as nano-carriers. To enable us to take advantage of these features, we have investigated the feasibility of *cyclo*-(d-Trp-Tyr) PNTs as an oral gene delivery carrier, and the results showed that PNTs and genes could distribute to duodenum, stomach, liver and kidney after the first oral PNTs. At 48–72 h after the first dose of oral delivery, the expression of delivered gene at both the protein and mRNA levels was also detected at those organs. Therefore, PNTs with a multifunctional nature appear to be required for ideal carrier capable for interacting with nucleic acid and penetrating the “tightly” organized epithelial layers of cornea, as well as to deliver DNA to cornea. In this study, we delivered the plasmid-encoding caspase 3 silencing shRNA with PNTs via eye drop to cornea with epithelial debridement and assessed its anti-apoptotic effect.

## 2. Experimental Section

### 2.1. Preparation of cyclo-(d-Trp-Tyr) Peptide Nanotubes

The self-assembly of *cyclo*-(d-Trp-Tyr) (Bachem, Bubendorf, Switzerland) peptide nanotubes (PNTs) was prepared according to our previous study with modification [25]. Briefly, 5 mg of *cyclo*-(d-Trp-Tyr) powder were dissolved in 1.5 mL of 50% ethanol in an Eppendorf tube. The Eppendorf tube was left opened, and a white suspension of nanotubes was obtained after equilibrating the gas phase for 48–72 h. Nanotubes were harvested by evaporation of residual ethanol.

### 2.2. Plasmid DNA

Caspase 3 silencing shRNA (CAP3 pRFP-C-RS) was purchased from OriGene Technologies (Rockville, MD, USA). CAP3 pRFP-C-RS constructs or control shRNA were transfected *in vivo* using PNTs as in our previous studies [16,25]. These plasmids were amplified in *Escherichia coli* host strain DH5α and purified by equilibrium centrifugation on a CsCl-ethidium bromide gradient [16,25]. The purity of the plasmid DNA prepared was determined by electrophoresis on an agarose gel followed by ethidium bromide staining. DNA concentration was measured by ultraviolet (UV) absorption at 260 nm [16,25].

### 2.3. Plasmid DNA Labeling

Plasmid CAP3 pRFP-C-RS was labeled with TM-rhodamine (Label IT^®^ nucleic acid labeling kit; Mirus, Madison, WI, USA) according to the manufacturer’s instructions, as described in our previous studies [25]. Briefly, CAP3 pRFP-C-RS was mixed with labeling buffer and labeling reagent. After incubating at 37 °C for 2 h, the labeled DNA was further purified by ethanol precipitation and confirmed by HPLC with a TSK-GEL^®^ G5000 PW_XL_ column (Tosoh Bioscience, Tessenderlo, Belgium) at a 0.7 mL/min flow rate with water (pH 5) as the mobile phase and detected by fluorescence detector (ex = 546 nm, em = 576 nm), as previously report [25].

### 2.4. The Formulation of Plasmid/PNTs Complexes

The CAP3 pRFP-C-RS/PNTs or TM-rhodamine-labeled CAP3 pRFP-C-RS/PNTs were formulated by gently mixing the plasmid DNA (0.08 μg/μL) with PNTs (0.15%, *w*/*v*) in an Eppendorf tube for 24 h at 25 °C, as previously described [25].

### 2.5. Characterization of CAP3 pRFP-C-RS/PNTs

#### 2.5.1. Scanning Electron Microscope Imaging

The PNT suspension was dropped on the mica surface and dried in a vacuum system. Samples were then coated with gold particles using the sputter coating method under vacuum of 2 millibar at 20 mA for 8 min and further observed by SEM. The SEM (S-2400/Hitachi instruments Inc., San Jose, CA, USA) was operated at an accelerating voltage of 3.0 kV.

#### 2.5.2. Atomic Force Microscope Imaging

Ten microliters of PNTs suspension were placed on a mica surface without further treatment, as in previous studies [25]. The AFM (diCPII; Digital Instruments/Veeco Metrology Group, Santa Barbara, CA, USA) was operated in a constant tapping mode. The cantilevers were standard NanoProbe silicon single-crystal levers (NSC15/AIBS; MikroMasch, Tallinn, Estonia). The constant force mode was used with a recommended scan frequency of 328 kHz. A scanner with a 5-μm scanning range was used, and all images were collected within a 5 × 5 μm^2^ area.

#### 2.5.3. Fluorescence Microscope Imaging

Ten microliters of TM-rhodamine labeled CAP3 pRFP-C-RS/PNTs complexes were placed on the slide surface and air dried. The labeled DNA and only thioflavin T-stained PNTs groups were imaged with a fixed exposure time by a fluorescence microscope (Olympus BX40, Tokyo, Japan).

#### 2.5.4. Size and Zeta Potential Measurement

The sizes of PNT (0.15%, *w*/*v*) suspensions and the Zeta potential of CAP3 pRFP-C-RS (0.08 μg/μL), PNTs alone and CAP3 pRFP-C-RS/PNTs complexes in water were measured by quasielastic laser dynamic light scattering (DLS) (Hydro 2000S and nano-series nano-ZS, respectively; Malvern Instruments, Malvern, UK), as described in our previous studies [16,25]. All measurements were performed at 25 °C at a measurement angle of 90° with an assumed refractive index ratio of 1.33.

#### 2.5.5. Fluorescence Measurement

In order to determine the association constant of the binding of Tyr in PNTs and the plasmid DNA, fluorescence measurements were performed following reports in other studies [25,26,27,28]. The emission spectra (emission slit 2.5 nm, F-4500 spectrophotometer, Hitachi instruments Inc., Tokyo, Japan) were measured upon excitation at 280 nm (excitation slit 2.5 nm), where both Trp and Tyr residues were excited, and at 295 nm, where only Trp residues were selectively excited. The binding constant K of Tyr to DNA was evaluated by the change of intensity in fluorescence emission spectra of PNTs in the presence of different concentrations of DNA excitation at 280 nm according to Equation (1) described in previous studies [25,27,28]:

(1) $$\log[\frac{F_{0}-F}{F}]=\log K+n\log\left[\text{DNA}\right]$$

Here, *F*_0_ and *F* are the fluorescence intensity of the fluorophore, Tyr, at 280 nm in the absence and the presence of different concentrations of DNA, respectively.

#### 2.5.6. Stability of CAP3 pRFP-C-RS /PNTs with DNase I

Protection of CAP3 pRFP-C-RS with PNTs against DNase I was carried out as described previously [16,25]. Briefly, 13 units of RQ1 RNase-free DNase I (Promega Biotech Co., Ltd, Madison, WI, USA) and 100 μg of CAP3 pRFP-C-RS with or without PNTs in a total volume of 200 μL were incubated at 37 °C. The mixture was sampled in each 10 μL of samples after incubating with DNase I at 37 °C for 0, 5, 10, 15, 20, 40, 55, 60, 65, 70 and 90 min, and then, 1 μL of RQ1 DNase I stop solution (Promega Biotech Co., Ltd, Madison, WI, USA) was immediately added to each sample. The resulting solutions were directly loaded onto a 0.8% agarose gel for electrophoresis, and then, the gel was stained with ethidium bromide. Qualification of band intensities was performed with a Kodak EDAS290 Analysis system (Kodak Scientific Imaging System, New Haven, CT, USA).

### 2.6. Animals Used for in Vivo Gene Delivery

The animal protocol was approved by the Laboratory Animal Research Committee of Taipei Medical University (NSC 101-2320-B-038-MY3 and MOST 104-2320-B-038 -014 -MY2). Male nude mice (BALB/cAnN-Foxn1nu/CrlNarl) at 6~8 weeks of age were used for epithelial debridement in cornea and *in vivo* eye drop delivery and were purchased from the National Laboratory Animal Breeding and Research Center (Taipei, Taiwan). They were maintained under specific pathogen-free conditions.

### 2.7. Epithelial Debridement in Mouse Cornea and Eye Drop Gene Delivery to Epithelial-Defective Cornea

Under general anesthesia and topical anesthesia, a round epithelial debridement of 2 mm in diameter was generated in the central cornea following previous reports [10,16]. After epithelial debridement injury of cornea, the injured eyes were confirmed by photographing with fluorescein (2%; Sigma Co., St. Louis, MO, USA) staining to assess the corneal epithelial debridement. After different periods of injury, the injured animals were euthanized, and the corneas with a 2 mm in diameter wounded area were recruited for further studies. For the *in vivo* eye drop delivery studies, plasmid at 0.08 μg/μL in 0.15% PNTs (10 μL per eye, six doses of eye drops and three times a day) was delivered to the eyes of mice immediately with or without corneal epithelial debridement [16]. The animals were euthanized at 48 h after the first topical administration, and the corneas in the wounded area were immediately removed for determining caspase 3 protein and its activity.

### 2.8. Distribution of CAP3 pRFP-C-R/PNTs in Mouse Epithelial-Defective Cornea

In order to trace the distribution of delivered DNA or PNT, the complexes of TM-rhodamine-labeled CAP3 pRFP-C-RS/thioflavin T-stained PNTs were administrated following the methods described in the section for eye drop gene transfer *in vivo* [16,25,29]. Mice also receiving no epithelial debridement were delivered only thioflavin T-stained PNTs. Mice were euthanized by cervical dislocation at 48 h after the first dose; cornea tissues were removed, and cryosections (10 μm) of the O.C.T.-embedded, paraformaldehyde-fixed eyes were washed for microscopy observation. After DAPI (1 µg/mL) staining for 20 min, sections were observed using confocal laser scanning microscope (Leica TCS SP5, Germany) with a diode (50 mW) and DPSS (diode-pumped solid state; 10 mW) laser light source.

### 2.9. Determination of Caspase 3 Activity

Caspase 3 activity was analyzed using the Caspase-Glo^®^ Assay (Promega Biotech Co., Ltd, Madison, WI, USA) with a modified protocol [16,30]. Briefly, cytosolic extracts of cornea tissues (2 mm) were prepared by dounce homogenization in hypotonic extraction buffer (25 mM HEPES, pH 7.5, 5 mM MgCl_2_, 1 mM EGTA and 1 μg/mL of leupeptin and aprotinin) and subsequently centrifuged to collect the supernatant. The protein concentration of supernatant was adjusted to 1 mg/mL with extraction buffer and stored at −80 °C. An equal volume of caspase-3 substrate and 50 μg cytosolic protein were incubated at room temperature for 1 h, and the caspase 3 activity was measured with a Veritas™ microplate luminometer (Turner BioSystems, Inc., Sunnyvale, CA, USA). Statistical comparisons were made with ANOVA tests with Dunnett’s multiple comparison tests at a 95% confidence level. All results were presented as the mean ± SEM.

### 2.10. Western Blotting Analysis

To analyze the protein level of caspase 3 after corneal epithelial debridement, the corneas in the wounded area of 2 mm in diameter at each time point were excised for Western blotting analysis [16]. Tissue homogenates were prepared by sonication with 2× SDS gel-loading buffer (100 mM Tris–HCl pH 6.8; 200 mM dithiothreitol; 4% SDS; 0.2% bromophenol blue and 20% glycerol); lysates were collected after centrifugation, and the protein concentrations were determined using the DC protein assay kit (Bio-Rad, CA, USA). Protein samples were separated on a SDS polyacrylamide gel and transferred to methanol-activated polyvinylidene difluoride (PVDF) membrane (Hybond-P; Amersham Biosciences, NJ, USA). Primary antibodies against caspase 3 (Santa Cruz Biotechnology, Inc., Paso Robles, CA, USA) at 1:200 dilutions and anti-β-actin antibody at 1:2000 as the loading control were incubated with the membrane. The membranes were then washed with 0.1% Tween 20 in Tris-buffered saline (TBS) and incubated with horse-radish peroxidase (HRP)-conjugated secondary antibody for 1 h at room temperature, followed by detection with enhanced chemiluminescence (ECL) analysis (ECL Western Blotting Detection Reagents, RPN2209; Amersham Biosciences, Hercules, NJ, USA).

## 3. Results and Discussion

### 3.1. Characterization of CAP3 pRFP-C-RS/PNTs

**Figure 1:**
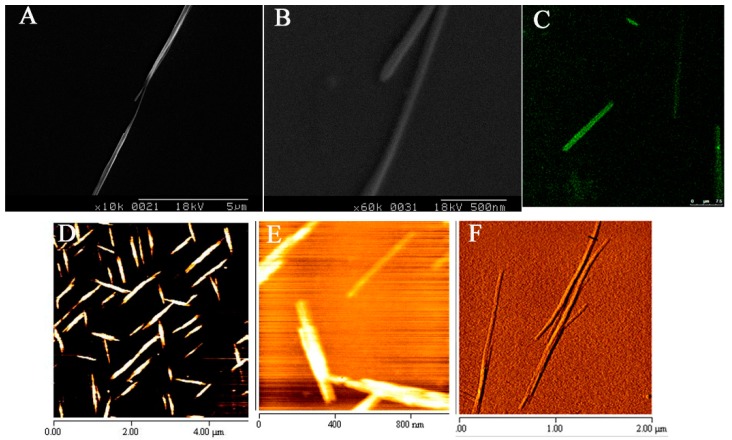
Morphology of *cyclo*-(d-Trp-Tyr) peptide nanotubes (PNTs). Images from scanning electron microscopy (SEM) (**A**,**B**), atomic force microscopy (AFM) (**D**–**F**) and staining with thioflavin T by fluorescent microscopy are presented (**C**).

The tube-shaped PNTs composed of *cyclo*-(d-Trp-Tyr) appeared to be 1.8 ± 0.6 μm in length as observed by scanning electron microscope (SEM) (Figure 1A,B). Images of AFM further found that *cyclo*-(d-Trp-Tyr) PNTs were tube shapes with 30–200 nm widths (Figure 1D–G). Using 0.2 mg of PNTs pre-staining with thioflavin T (4 μM), a dye that has been used to stain PNTs [25,29,31], the resulting PNTs are shown to be similar (Figure 1C) to unlabeled PNTs. The labeled thioflavin T with PNTs showed a similar nanotube shape with a comparable length and width range reported by others using diketopiperazine *cyclo*-dipeptide with 50% methanol as the solvent [32,33,34]. They reported that the widths or shapes of fiber were influenced by different solvents and their peptide properties, which could affect or even induce aggregated ensembles.

**Figure 2:**
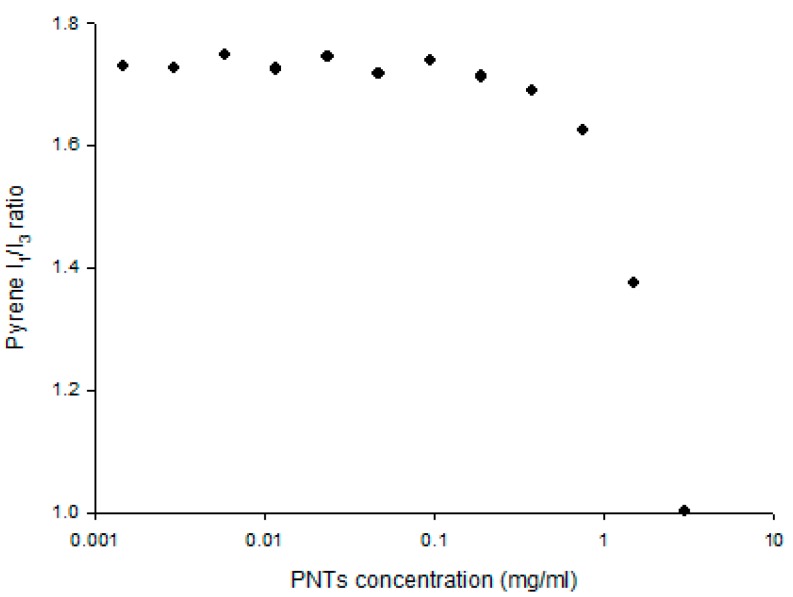
Self-association of *cyclo*-(d-Trp-Tyr) peptide nanotubes (PNTs). The self-association property was estimated by a pyrene fluorescence probe. For the pyrene solution, 6.7 × 10^−7^ M was used, and the critical association concentration (CAC) was determined by the turning point of *I*_1_/*I*_3_ ratio represented as a solid circular symbol.

The self-association of PNTs was evaluated using pyrene as the fluorescence probe. The critical association concentration (CAC) was determined by the pyrene *I*_1_/*I*_3_ ratio, a well-known property reflecting the microenvironment polarity [16,25,35]. Results showed that the CAC of PNTs was above a 0.1 mg/mL concentration (Figure 2), which is similar to our previous reports [25]. To further evaluate the formation of PNTs, the sizes of PNTs above the CAC of 1.5 mg/mL were analyzed by quasielastic laser dynamic light scattering (DLS). The overall size of PNTs at a 1.5 mg/mL concentration was an average of 2.2 ± 0.5 μm measured by DLS (Table 1), which was similar to the length estimated on the images obtained by fluorescent and SEM microscopes. To ensure that PNTs remained in a tubular shape, PNTs at this concentration (1.5 mg/mL) were used for all further *in vivo* eye drop delivery.

**Table 1:** Size and Zeta potential of CAP3 pRFP-C-RS (P) formulated with *cyclo*-(d-Trp-Tyr) peptide nanotubes (PNTs).

Formulation	DLS Size (μm) ^a^	Microscope ^b^		ζ-potential (mV) ^c^	
		Width (μm)	Length (μm)		
P ^d^	0.068 ± 0.003	–	–	−47.1	±7.7
PNTs ^e^	2.2 ± 0.5	0.29 ± 0.08	1.8 ± 0.6	−37.6	±2.8
P/PNTs ^f^	1.7 ± 0.6	0.26 ± 0.06	1.4 ±0.8	−74.3	±1.3

^a^ Results are expressed as the mean and standard deviation (mean ± SD) for six experiments; ^b^ values represent the range of particle sizes measured by matching the scale bar visually in SEM images; ^c^ results are expressed as the mean ± SD for three experiments; ^d^ CAP3 pRFP-C-RS (0.08 mg/mL); ^e^ PNTs (0.15%); ^f^ CAP3 pRFP-C-RS (0.08 mg/mL) formulated with PNTs (0.15%) (P/PNTs).

**Figure 3:**
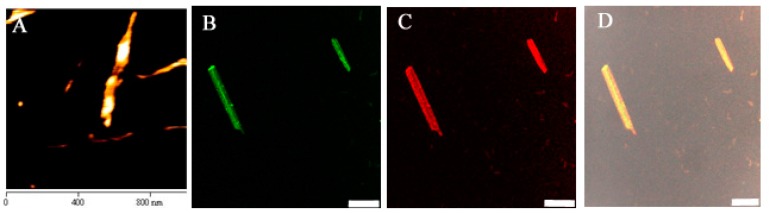
Atomic force microscope (**A**) and fluorescence microscope imaging of TM-rhodamine-labeled CAP3 pRFP-C-RS image (red color) (**C**) formulated with *cyclo*-(d-Trp-Tyr) ThT pre-stained peptide nanotubes image (green color) (**B**). (**D**) The imaging was merged with (**B**) and (**C**). Scale bars denote 2.5 μm.

Furthermore, the overall size of CAP3 pRFP-C-RS/PNTs formulation was an average of 1.7 ± 0.6 um measured by DLS (Table 1), and this was similar to the length of the PNT formulation observed by SEM. The similar size distribution of PNTs and CAP3 pRFP-C-RS/PNTs suggested that the presence of plasmid DNA may not affect the sizes of PNTs. To further analyze the effect of DNA on the surface charge, the Zeta potential of the CAP3 pRFP-C-RS/PNTs formulation was measured. The results (Table 1) revealed that the Zeta potential of CAP3 pRFP-C-RS or PNTs alone in water was −47.1 ± 7.7 and −37.6 ± 2.8 mV, respectively. However, the Zeta potential was shifted to −74.3 ± 1.3 mV when CAP3 pRFP-C-RS was formulated with PNTs, indicating that the plasmid DNA might associate on the surface of PNTs. To further confirm the association of DNA on the surface of PNTs, TM-rhodamine-labeled CAP3 pRFP-C-RS was also associated with PNTs and detected by fluorescence microscope (Figure 3), as well as imaging with AFM.

**Figure 4:**
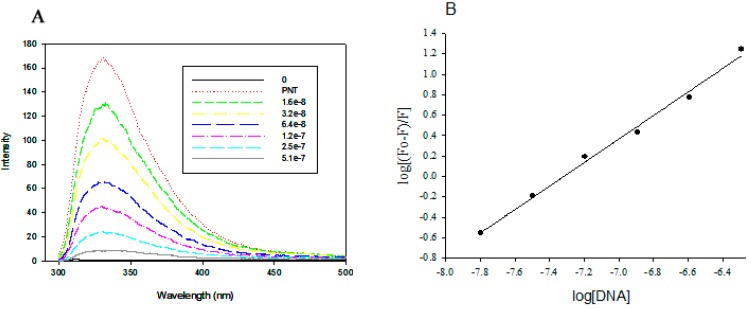
Fluorescence quenching assay of *cyclo*-(d-Trp-Tyr) peptide nanotubes (PNTs) with CAP3 pRFP-C-RS. (**A**) The emission fluorescence spectra of PNTs at 1.6 × 10^−^^8^ M upon binding to various concentration of DNA (1.6 × 10^−^^8^, 3.2 × 10^−^^8^, 6.4 × 10^−^^8^, 1.2 × 10^−^^7^, 2.5 × 10^−^^7^, 5.1 × 10^−^^7^ M) with excitation at 280 nm for the detection of fluorescence from both of Tyr and Trp residues. (**B**) The linear plot for log(*F*_0_ − *F*)/F *vs.* log[DNA] according to Equation (1) with *r*^2^ = 0.9968.

In order to confirm the involvement of Tyr residues of PNTs in association with DNA, the fluorescence emission spectra of PNTs with or without DNA were examined. The emission intensity contributed by Tyr of PNTs (Figure 4A), with excitation at 280 nm [21], was significantly decreased when DNA was added, and the results were similar to our previous studies [25]. The emission intensity of fluorescence with excitation at 295 nm, which was specific for Trp [21] in PNTs, however, was not influenced by the addition of DNA (data not shown) [25]. The quenching at Tyr fluorescence emission spectra was found to be augmented with the increasing concentration of DNA used. The binding constant (K) of Tyr residues in PNTs to DNA and the mole fraction of bound DNA were calculated to be 8.43 × 10^8^ M^−1^ and 1.15 mole fraction of DNA bound to Tyr, respectively (Figure 4B). These were supported by another report that PNTs with a neutral amino acid, Tyr, were able to associate with DNA by stacking the phenolic oxygen of Tyr between the AT pairing in the DNA double-helix via an electron-transfer interaction [26]. Several studies also confirmed that Tyr in both linear and cyclic peptides stacked its aromatic ring between the base pairing of the DNA molecule [26,32]. In addition, it was reported that the binding constant of short peptides, such as linear Lys-Tyr-Lys or *cyclo*-(-Lys-Tyr-Lys-Ahx-) with DNA, was estimated to be far below 1 × 10^3^ M^−1^ [36]. Therefore, different ratios between DNA and PNTs, as well as the lengths of PNTs not only could influence the binding/releasing pattern of DNA, but also may affect the internalization rates, penetration behavior and stability during circulation due to their different aspect ratios (AR) [17,18,19].

### 3.2. Stability of CAP3 pRFP-C-RS/PNTs with DNase I

**Figure 5:**
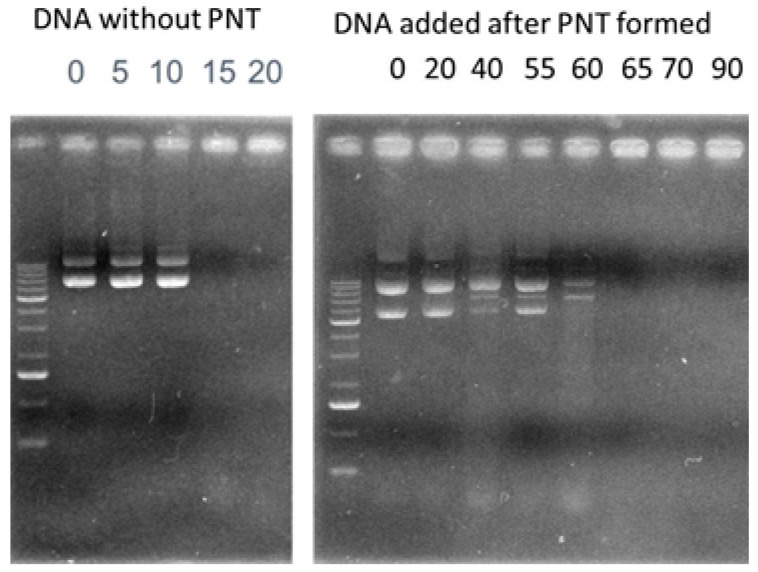
Stability of CAP3 pRFP-C-RS (7.4 kb) with *cyclo*-(d-Trp-Tyr) peptide nanotubes (PNTs) analyzed by DNase I. Samples treated with DNase I and CAP3 pRFP-C-RS added after PNTs formed at each time point were electrophoresed on 0.8% agarose gel. The markers (M) represented DNA of 10, 8, 6, 5, 4, 3, 2.5, 2, 1.5, 1, 0.75, 0.5 and 0.25 kb in size. At the 0 min time point of the reaction performed without DNase I, supercoil and multi-mer forms of DNA were detected.

We next analyzed the *in vitro* stability of DNA in the formulation with PNTs with DNase I [16,25]. Results showed that naked DNA was completely digested soon after incubation with DNase I at 37 °C within 10 min (Figure 5). The supercoiled CAP3 pRFP-C-RS with a size of 7.4 kb was observed after DNase I digestion for 60 min in the form formulated with PNTs. The 60 min-delayed degradation was also found using carbon nanotubes [37], as well as in our previous studies [25].

### 3.3. In Vivo Eye Drop Gene Transfer in Cornea Area

**Figure 6:**
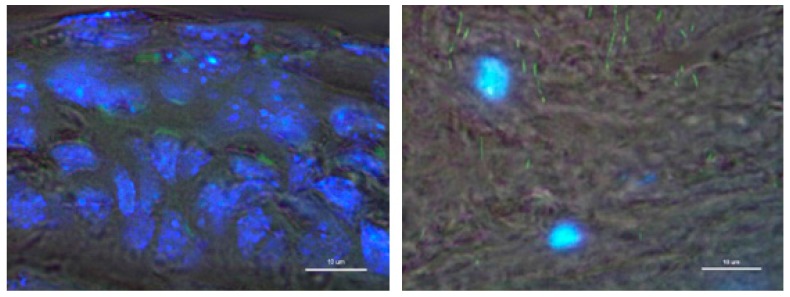
The histological analysis of *cyclo*-(d-Trp-Tyr) peptide nanotubes’ (PNTs) distribution in tissues of nude mice after 180 min of eye drop delivery with thioflavin T (ThT) pre-stained PNTs. The ThT image (green) is merged with DAPI nuclear staining (blue) and the bright field image. Note that the ThT pre-stained PNTs (green color) were located in both the epithelial (left) and stroma area (right). Scale bars denote 10 μm.

In order to investigate the feasibility of this PNT for being a useful eye drop gene delivery carrier, mice were administrated eye drops with thioflavin T (ThT)-stained PNTs. The PNTs were found in both regions of the epithelial and stroma area after 180 min (Figure 6), indicating the presence of PNTs that could penetrate into cornea areas. Although our previous study showed a decrease in both length and width of PNTs detected over 100 min in the presence of simulated gastric acid at pH 2 [25], the small sized PNTs could distribute in four major organs, including stomach, duodenum, liver and kidney after 1 h of oral delivery with thioflavin T (ThT) pre-stained PNTs. Furthermore, Jiban *et al.* [38] reported that some intact dipeptide nanotubes were still present in the vitreous humor of eye at the end of a 15-day period by intra-vitreal injection. However, since cornea consists of epithelial layers, which are a major rate-limiting barrier to drug absorption, the collagenous stroma layer with hydrophilic properties, as well as an internal endothelium [39,40,41], Zang *et al.* demonstrated that a peptide of 27 amino acid residues forming an ion channel could transiently open the intact epithelial barrier to allow the permeation of small molecules into the stroma [41]. They found that in the presence of their peptide at corneal stromal depths around 50, 100 and 150 μm was significantly high and almost as high as in de-epithelialized corneas. They therefore proposed that the mechanisms for their peptide entering cornea epithelium may be different from those of EDTA opening the tight junction of epithelium to allow the diffusion of small molecules. We showed in our previous *in vitro* duodenal permeability studies that the penetration of *cyclo*-(d-Trp-Tyr) peptide PNTs formulated with DNA was energy and directionally dependent [25]. Therefore, the penetration mechanisms of PNTs through cornea epithelium still need to be further investigated systemically to elucidate all of the potential factors.

### 3.4. Gene Delivery via Eye Drop to Mouse Cornea after Epithelial Debridement

**Figure 7:**
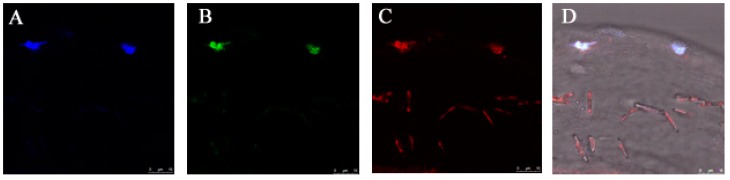
The histological observation of *cyclo*-(d-Trp-Tyr) peptide nanotubes (PNTs) with the TM-rhodamine-labeled plasmid CAP3 pRFP-C-RS distribution in tissues of nude mice after 8 h of eye drop delivery with thioflavin T (ThT) pre-stained PNTs. DAPI image (blue) (**A**), ThT image (green) (**B**), rhodamine image (red) (**C**) merged with the bright field image (**D**). Note that the small ThT pre-stained PNTs (green color) and rhodamine-labeled plasmid (red) were co-located in the stoma area of cornea. Scale bars denote 10 μm.

**Figure 8:**
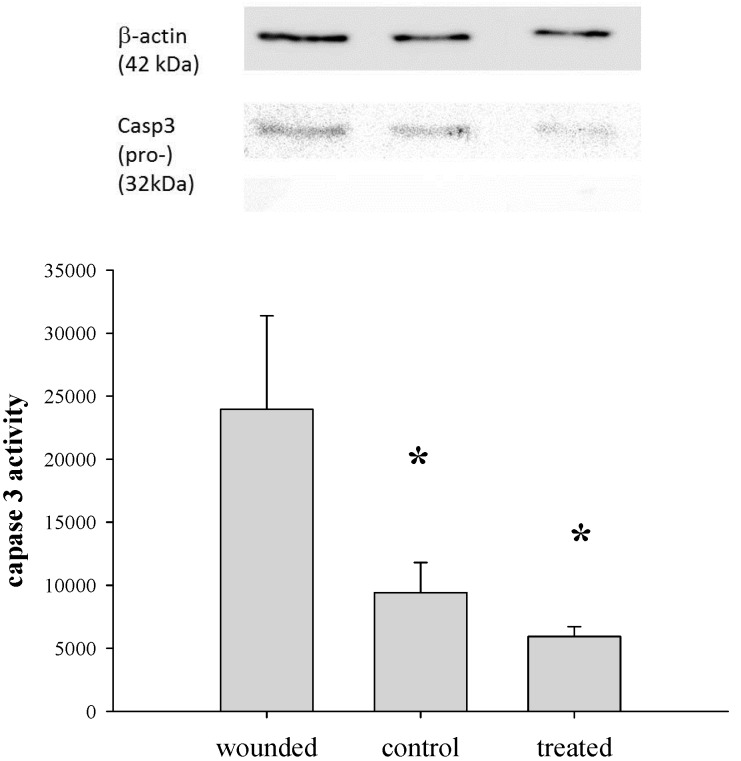
Caspase 3 activity of CAP3 pRFP-C-RS/PNT delivery in cornea after epithelial debridement. The caspase 3 activity was significant decreased at 48 h of CAP3 pRFP-C-RS/PNT delivered to epithelial-debrided cornea compared with the same time after debridement without treatment. * Significant difference (*p* < 0.05).

The injured corneas were first confirmed by photography with fluorescein before gene delivery with PNTs. After corneal epithelial debridement, the anti-apoptotic CAP3 pRFP-C-RS/PNTs was delivered, and a 2-mm diameter section within the central debridement area of the cornea was excised for detecting labeled plasmid DNA formulated with thioflavin T pre-stained PNTs. The results showed that after one dose of eye drop of CAP3 pRFP-C-RS/PNTs to the wounded cornea, the PNTs and DNA were found distributed within the stroma region (Figure 7). This was consistent with the observation of Pescina *et al.* that the diffusion of oligonucleotides was not hindered by corneal stroma after being de-epithelialized [40]. Although our previous study showed that the release rate of DNA in the PNT formulation was in a slow release process [25], we detected some of the labeled plasmid DNA distributed around the nuclear area of the keratocyte cell. After 48 h after the first dose of the eye drop of CAP3 pRFP-C-RS/PNTs, mice were euthanized, and the caspase 3 activities in cornea were evaluated. The results (Figure 8) showed that the caspase 3 activity significantly decreased in cornea (41%) at 48 h after the first dose of the eye drop administration of pRFP-C-RS/PNTs (*p* < 0.05). The protein level of caspase 3 was also found to be decreased in wounded corneas after six doses within 48 h of the eye drop of CAP3 pRFP-C-RS/PNTs, which is comparable to our previous studies with pCMV-bcl-x_L_-eGFP/polymeric micelle delivery after cornea debridement. Although faster internalization was reported for nanotubes with a higher aspect ratio (AR) than spherical ones [17,18,42], the observed similar decreased caspase 3 level could be due to the removal of the major transport barrier of the epithelial layer, and corneal stroma, therefore, could not hinder any hydrophilic compounds. In addition, with delivery of the same pCMV-bcl-x_L_-eGFP plasmid formulated with PNTs, we found a similar decreased level of caspase 3 (data not show). In summary, our results provide evidence showing the feasibility of this PNT to penetrate the intact cornea via eye drop delivery and to deliver CAP3 pRFP-C-RS DNA to decrease the apoptotic protein triggered by corneal epithelial debridement. Reducing apoptosis was successfully detected in the wounded cornea after delivery.
